# The Versatile Role of miR-21 in Renal Homeostasis and Diseases

**DOI:** 10.3390/cells11213525

**Published:** 2022-11-07

**Authors:** Romain Larrue, Sandy Fellah, Cynthia Van der Hauwaert, Marie-Flore Hennino, Michaël Perrais, Arnaud Lionet, François Glowacki, Nicolas Pottier, Christelle Cauffiez

**Affiliations:** 1 Univ. Lille, CNRS, Inserm, CHU Lille, UMR9020-U1277—CANTHER—Cancer Heterogeneity Plasticity and Resistance to Therapies, F-59000 Lille, France; 2 CHU Lille, Département de la Recherche en Santé, F-59000 Lille, France; 3 CH Valenciennes, Service de Néphrologie, F-59300 Valenciennes, France; 4 CHU Lille, Service de Néphrologie, F-59000 Lille, France

**Keywords:** microRNA, kidney, cancer, fibrosis

## Abstract

MicroRNAs (miRNAs) are small, non-coding RNA species that control gene expression and confer robustness to biological processes. Over the last two decades, their important roles during kidney development, homeostasis and the treatment of diseases have been established, in particular during the onset and progression of various forms of acute and chronic renal disorders. In recent years, miR-21, one of the best-characterized miRNAs to date, has received much attention in renal physiology in particular given its high degree of conservation and expression in kidneys, as well as its potent pathogenic role in various debilitating renal diseases. This review summarizes the current knowledge on miR-21’s involvement in both renal homeostasis and diseases, in particular its double-edged-sword role in acute versus chronic kidney injuries. Finally, we also discuss the potential of miR-21 as a biomarker and therapeutic target in renal diseases.

## 1. Introduction

More than 2500 microRNAs (miRNAs) are expressed in human cells (miRbase v22.1). Some of them exhibit tissue- or cell-specific expression patterns, while others are considered as house-keeping molecules [1]. Although miRNAs were discovered in the early 1990s through the analysis of developmental timing mutants in *C. elegans* [2,3], their biological significance was underestimated until 2001, with the identification of numerous endogenously expressed small RNAs in worms, flies and mammals [4,5,6]. We have since learned that miRNAs represent critical gene expression regulators that control major cellular functions in various physiological and pathological settings [7]. Although a single miRNA potentially regulates the expression of multiple protein-coding genes, it is now clear that not all miRNAs are equally important. Indeed, lessons from various research studies have identified a limited number of key functional miRNAs such as miR-21, one of the most extensively studied miRNA [8]. Since its initial description in 2005 as the miRNA most commonly and strongly up-regulated in human brain tumor glioblastoma [9] miR-21 has received considerable attention, given its consistent upregulation and pathogenic role in multiple distinct disease conditions. In this review, we summarize the current knowledge of miR-21’s functions in kidney diseases, with emphasis on its potential as a disease biomarker and novel therapeutic target. For easier reading, miR-21 will be used throughout the manuscript instead of hsa-miR-21-5p or mmu-miR-21a-5p.

## 2. miR-21’s Genomic Organization, Biogenesis and Regulation

**Figure 1:**
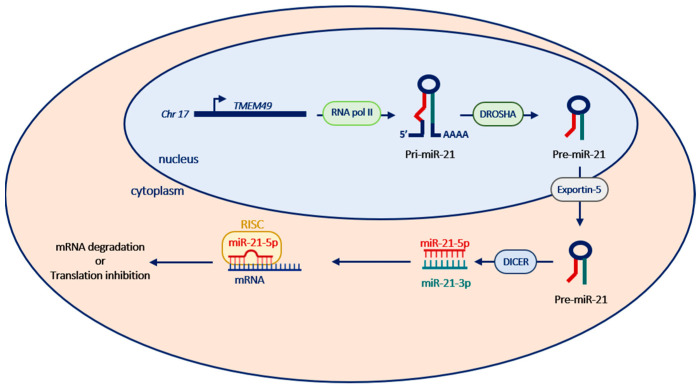
Biogenesis of miR-21. miR-21 is transcribed by RNA polymerase II from chromosome 17 into pri-miR-21 transcript, which is then processed by a microprocessor into hairpin precursor molecules (pre-miR-21) in the nucleus. These are exported to the cytoplasm by exportin 5 and are further processed into mature miR-21 sequences, which are then incorporated into the RISC and guided to miR-21 target mRNAs to repress their expression.

In contrast with most miRNAs which usually map in intergenic or intronic regions of coding or non-coding genes, the mature sequence of miR-21 is located immediately downstream of the 3′-unstranslated region of the coding gene VMP1 (Vacuole Membrane Protein 1), also known as TMEM49 (Transmembrane Protein 49) [10]. The gene encoding miR-21 is transcribed by RNA polymerase II independently of VMP1 into a capped, polyadenylated and unspliced primary transcript (pri-miR-21) and then processed by the RNAse III enzyme Drosha to form an approximately 70 nucleotide precursor miRNA (pre-miR-21). The pre-miR-21 is then shuttled into the cytoplasm and forms the mature miRNA of 22 nucleotides following processing by a second RNAse III enzyme called DICER. The mature miR-21 is then incorporated into the RNA-induced silencing complex (RISC), which is able to recognize the “seed sequence” of the miRNA in target mRNA and negatively regulate its expression (Figure 1) [10].

**Figure 2:**
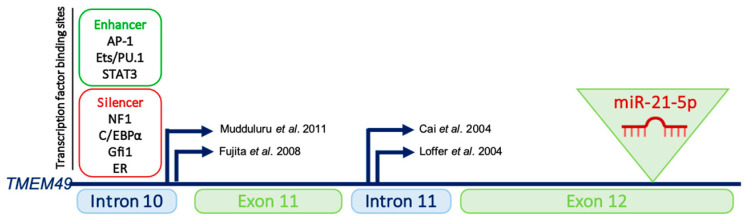
Genomic location of pri-miR-21. Gene encoding pri-miR-21 is located on chromosome 17q23.2 and overlaps with TMEM49, a coding gene. Two distinct transcriptional start sites with respect to the miR-21 hairpin have been described and are indicated by bent arrows [13,14,15,16]. Binding regions for transcriptional activators (AP-1, Ets/PU,1 and STAT3) and repressors (NFI, C/EBPα, Gfi1 and ER) are also shown.

Like any other gene product, miRNAs are subject to transcriptional regulation, a fundamental process controlling gene expression [11]. Transcriptional regulation involves the complex interplay of genomic cis-regulatory elements, transcription factors, co-activator/co-repressor complexes, chromatin modifications and other epigenetic factors (Figure 2) [12].

Although notable exceptions exist, intergenic miRNAs are known to be transcribed as independent transcription units while intronic miRNAs are believed to be processed from the introns of their hosting transcription units, and hence share common regulatory mechanisms and expression patterns with their host gene [17]. MiR-21 is one of the very few miRNAs which have been mapped in the 3′-untranslated regions (UTRs) of a coding gene. It is indeed located immediately adjacent to the 3′UTR of the TMEM49 gene [12]. While it was initially assumed that miR-21 was expressed as part of the TMEM49 transcript, many studies have instead experimentally shown that miR-21 and its host gene are independently regulated, and that local promoter regions initiate miR-21 transcription through long, unspliced and non-coding pri-miR-21 transcripts [18]. A detailed analysis of the upstream region of miR-21 identified several highly conserved enhancer elements, including binding sites for AP-1, Ets/PU.1 or STAT3. Experiments using heterologous luciferase have notably revealed that the PMA (phorbol myristate acetate)-induced activation of AP-1 or STAT3 activation results in the transcription of pri-miR-21 [8]. Given that these two transcription factors have well-recognized roles in tumorigenesis, up-regulated miR-21 expression in cancers may in fact reflect their aberrant tumoral expression. Furthermore, several transcriptional suppressors have also been reported, such as NF1, C/EBPα, Gfi1 and ER, indicating that miR-21 transcriptional levels are rather controlled by the balance between stimulatory and inhibitory transcription factors [19].

Epigenetic modification is another mechanism modulating miR-21 expression. For example, Baer et al., showed that the upregulation of miR-21 in chronic lymphocytic leukemia is linked to the DNA methylation of the miR-21 upstream sequence. As a matter of fact, the miR-21 promoter sequence was completely unmethylated in all leukemia samples but showed significant DNA methylation in controls [20]. Similarly, Iorio et al. demonstrated that miR-21 is strongly induced in ovarian cell line OVCAR3 treated with the demethylating agent 5-AZA [21].

In addition to transcriptional regulation, Davis and colleagues have shown that miR-21 is also subjected to post-transcriptional regulation. Indeed, they demonstrated that miR-21 expression can be induced through a SMAD-mediated increase in the Drosha processing of the pri-miR-21 transcript, and further showed that this novel regulatory mechanism is not only critical for the control of the vascular smooth muscle cell phenotype but also has functional consequences in the promotion of metastasis [22].

Mature miR-21 activity has been reported to be linked in vivo to its differential association with polysomes that reflects distinct strengths of interactions with mRNA targets according to the cellular context. For example, whereas miR-21 displays poor association with polysomes in healthy mouse hepatic tissue, associated with a low translation repression [23], a strong association of miR-21 with polysomes in HeLa cells has been shown to be related to high inhibition activity in this context [24]. A rapid and reversible regulation of miR-21 can also be mediated by a sequestration mechanism of mature miR-21 copies. Androsavitch et al. have shown that the miR-21-mRNA interaction is thermodynamically unstable due to a high adenine and uracil content within the seed sequence [23], resulting in a high sensitivity of miR-21 to “miRNA sponge” molecules that can prevent miRNA from binding to its targets. Endogenous “miRNA sponges” have previously been described. For example, GAS5 lncRNA, which has been identified as a novel target for miR-21, also contains a putative miR-21 binding site, resulting in the negative regulation of miR-21 [25,26]. In addition, pseudogenes frequently include nonsense mutations located at the beginning of the coding sequence, resulting in a short transcript that will not be supported within the polysomes. Pseudogenes-sequestered miRNAs are then located outside of the polysomes, and remain inactive. This mechanism can be illustrated by the pseudogene PTENP, which is targeted by PTEN-targeting miRNAs and could then contribute to miR-21 regulation through its sequestration [27]. Synthetic “miRNA sponges” have already been extensively used for experimental purposes in the last decades [28,29,30,31,32].

## 3. miR-21 in Kidney Development and Normal Tissue

### 3.1. miR-21 in Kidney Development

miRNAs are critically implicated in the development of various organs [33], including kidneys, as demonstrated by the Dicer1 conditional deletion in renal progenitor cells that results in profound defects in nephrogenesis, characterized by a very severe nephronic reduction phenotype associated with increased apoptosis, architectural defects and 36 hr postpartum mouse mortality [34]. Moreover, Dicer conditional deletions in cortical stroma cells, podocytes and juxta-glomerular cells lead to various nephrogenesis anomalies affecting renal cells, including podocytes and tubular cells or microvascularizations [35,36,37,38].

In the zebrafish model, miR-21 expression has been reported at very-early stages of development and in relatively high amounts (up to 40% of all miRNAs in fibroblasts) [39]. However, miR-21 knock-out mice do not exhibit any pathological phenotype with normal viability and fertility, suggesting that miR-21 is not essential for normal development [40]. In particular, the mouse renal ultrastructure was strictly normal at 12 weeks of age [41].

### 3.2. miR-21 in Renal Regeneration

Whereas the number of mature nephrons is defined at birth in mammals, lower vertebrates (and especially fish) display nephrogenesis abilities in response to renal aggression [42]. It has been shown in particular that after renal injury, a population of progenitor cells is activated to de novo regenerate new nephrons, characterized by the presence of basophilic cells [43]. Interestingly, using the short-lived killifish *Nothobranchius furzeri* model, Hoppe et al. observed an increase in miR-21 renal expression after gentamicin-induced renal injury [44]. Moreover, prior administration of a miR-21 antagonist delayed renal recovery. Gene ontology analysis reveals that most of the genes whose expression was modulated by miR-21 antagonism were linked to apoptosis at an early stage or to cellular reorganization (i.e., membrane invagination or cellular homeostasis) at a later stage [44].

### 3.3. miR-21 in Normal Tissue

miR-21-5p is recognized as one of the most abundant and highly conserved miRNAs. In particular, the distribution of miRNA expression across human tissues according to the “human miRNA tissue atlas” database shows its ubiquitous expression at high levels in most tissues (https://ccb-web.cs.uni-saarland.de/tissueatlas, accessed on 31 October 2022). Whereas miR-21 is one of the most highly abundant miRNAs in tissues [8], its role under normal cellular conditions is not well understood. Indeed, following the pharmacological inhibition or genetic deletion of miR-21 in a healthy mouse, only a limited number of genes are deregulated in normal and unstressed tissues [40,45]. Moreover, a limited transcriptomic response (i.e., a lack of gene derepression) to miR-21 inhibition was observed in the livers of wild-type animals under basal conditions, as mRNA targets were not enriched for seed sequence matches. Interestingly, stress-response genes were however significantly enriched, particularly Taf7, a TBP-associated factor [23]. Compared with other abundant miRNAs in normal tissues, miR-21 also displays a reduced ability to bind to polysome-associated target mRNAs, and intriguingly, the derepression of the well-known miR-21 target Pdc4 was not observed [23]. Under similar conditions, miR-21 has been described to be poorly efficient in the lungs and heart [45,46].

Taken together, these observations suggest that, under normal cellular conditions, miR-21 activity is maintained below a threshold required for binding and silencing most of its targets.

## 4. miR-21 in Kidney Injuries and Diseases

In contrast with normal kidney function, miR-21 switches to a powerful and overactive mediator under stress conditions [23]. In particular, miR-21 is one of the most highly upregulated miRNAs in a wide panel of tissue injuries, and may act as a cellular sensor of injuries that mediates tissue regeneration.

### 4.1. miR-21 in Acute and Chronic Kidney Diseases

miR-21 has been associated with the development of a large number of both acute and chronic renal diseases. The main studies investigating the contribution of miR-21 in human renal disorders are summarized in Table S1. These studies consistently report a ubiquitous and non-specific increase of miR-21 renal expression in both acute and chronic renal diseases. Numerous experimental models have also explored the role of miR-21 using animal models of acute renal failure or chronic kidney diseases (Table S2).

#### 4.1.1. A Protective Role of miR-21 in Acute Kidney Injury?

Acute renal diseases are mainly represented by acute tubular necrosis (ATN), whose causes, although variable, are often related to two main mechanisms: ischemia (induced by hypovolemia, hemorrhage...) and iatrogeny (aminoglycosides, iodine, renin-angiotensin system blockers, cisplatin...) [47]. The evaluation of renal miR-21 expression in clinical samples remains patchy, due to the fact that renal biopsies are rarely performed, except for in the context of renal transplantation [48]. ATN is mainly associated with an increased miR-21 level in renal tissue, serum, or urine of patients with ATN [48,49,50,51]. Furthermore, a large number of ATN-mimicking animal models rely on ischemia-reperfusion mouse models [40,49,52,53,54,55,56,57,58,59,60] or the administration of nephrotoxic compounds, mainly gentamycin [49,55] or cisplatin [61]. All of these models demonstrated an early increase of miR-21 renal expression [58] that may be prolonged up to 30 days after injury [52]. Following repeated low-intensity injuries, miR-21 increase could thus initially plays a protective role, inducing wound healing and tissue regeneration processes by targeting PTEN [51], PDCD4 [53], PHD2 [60], the MKK3–MAPK–p38 pathway [58], thromospondin-1 [59] and Rab11A [56]. However, there is conflicting evidence regarding the protective or deleterious nature of miR-21. Indeed, in most studies, the inhibition of miR-21 leads to histological damage worsening and decreased renal function [53,55,56] after ischemia reperfusion, even if a protective preconditioning intervention, such as cobalt chloride injection [60], Xenon inhalation [55] or ischemic preconditioning [53], was beforehand applied. By contrast, Chau et al. reported that miR-21 inhibition improves histological injuries and albuminuria seven days after ischemic injury [40]. The main elements that may explain the difference between those studies are the variable miR-21 inhibitor injection schedule and endpoints (in particular euthanasia delay post-injury). We can thus hypothesize that miR-21 plays a protective role at the early stage of ischemia-reperfusion lesions, in particular in preconditioning interventions, but plays a secondary deleterious role once ATN lesions have been initiated.

#### 4.1.2. Sustained and Persistent Expression of miR-21 Has a Deleterious Impact in Chronic Kidney Diseases

miR-21 has been shown to be elevated in renal tissue, blood or urine in clinical samples from various pathologies (Table S1). As is consistent with most studies, a miR-21 increase is associated with more severe damages [41,62,63,64,65,66,67,68]. Similarly, an increased expression of miR-21 is unanimously reported in a plethora of chronic kidney disease mouse models (Table S2), underlining the deleterious role of miR-21 in chronic kidney diseases, including diabetic nephropathy [69,70,71,72,73], unilateral ureteral obstruction [40,69,74] and Alport syndrome [75].

**Figure 3:**
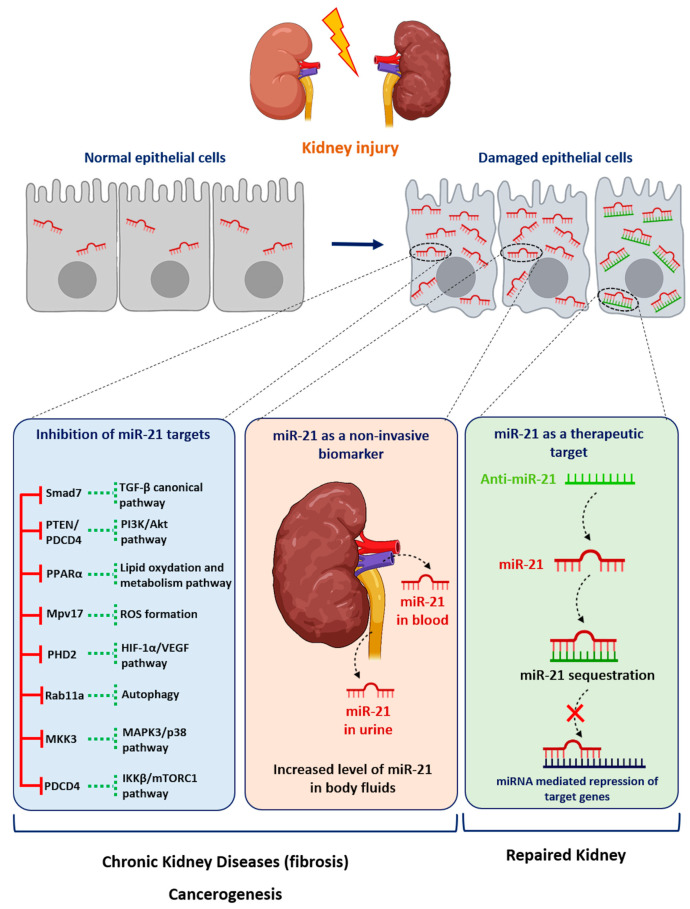
Role of miR-21 in kidney diseases. Following injury, the expression of miR-21 is increased in renal cells. miR-21 promotes kidney diseases by repressing various target genes. Levels of miR-21 can be assessed in urine or blood samples as a biomarker of kidney injury. Given its established pathogenic role in kidney disorders, targeting miR-21 using antisense oligonucleotides may represent a new therapeutic strategy for renal diseases.

It is noteworthy that most disparate diseases, such as diabetes mellitus, hypertension, Alport Syndrome or acute renal injuries, result in the development of either glomerular or interstitial fibrosis. Converging evidence from computational, biochemical and genetic experiments has indeed shown that miR-21 is a genuine profibrotic miRNA, regardless of the injured organ. In particular, miR-21 is invariably upregulated during the fibrogenic response to tissue injury and promotes the TGF-β signaling pathway, the major driver of tissue fibrosis [76]. In particular, Chau et al. produced a miR-21-null mouse to investigate the role of this miRNA in kidney fibrosis [40]. As is consistent with previous findings reported in cardiac [77] and lung fibrosis [78], injured kidneys from miR-21-deficient mice exhibited less fibrosis. Unexpectedly, the authors further showed that miR-21 primarily regulates the genes involved in lipid metabolism and mitochondrial redox regulation, rather than genes implicated in matrix turnover, inflammation or innate immunity. Indeed, the authors identified PPAR-α, a major transcription factor that regulates a number of lipid oxidation and metabolism pathways, and Mpv17l, which is thought to inhibit ROS formation by mitochondria, as direct targets of miR-21 [40]. This distinct mechanism was explained by the identification of epithelial cells as the major cellular source of increased miR-21 expression. Of particular interest, this study highlights that miR-21 can drive fibrogenesis by several distinct mechanisms, depending on the cellular context (Figure 3).

### 4.2. miR-21 as an “oncomiR”

miRNAs influence numerous cellular processes, including cell cycle regulation, differentiation and apoptosis, and can therefore act as either tumor suppressors or oncogenes [79]. Consequently, alterations in miRNA gene expression have a major impact on tumorigenesis. In particular, the overexpression of miR-21 is associated with many forms of cancer, and functional studies have established this miRNA as a genuine oncomiR (Figure 3). Indeed, many studies have demonstrated that miR-21 has a central role in tumor initiation and progression by targeting critical tumor suppressive genes, such as PTEN or PDCD4. Not surprisingly, comprehensive studies assessing miRNA expression in renal cell carcinoma (RCC) have shown widespread miRNA dysregulation, with many of these aberrantly expressed miRNAs targeting components of key oncogenic networks associated with RCC, including the HIF-, TGF-β- or mTOR-signaling pathways. Several studies on RCC have shown that miR-21 is overexpressed in the clear cell (cRCC) and papillary (pRCC) subtypes of RCC tumors compared with healthy kidneys and benign renal tumors [79,80,81,82,83,84,85]. In cRCC tissue patients, there is no relationship between miR-21 expression and age, laterality or gender [84,85]. Chen et al. have shown, in a cohort of 104 RCC tissue samples, that a higher miR-21 level is associated with larger tumor size, more lymph node metastasis and an advanced TNM stage [85]. In contrast, another study has shown in a cohort of 99 cRCC tissue samples that miR-21 expression was not associated with stage, nuclear Fürhman grade nor patient outcome [84]. Thus, miR-21 expression alone in primary tumors seems of limited interest as a diagnostic or a prognostic biomarker, and should rather be included in miRNA signature [86,87,88,89]. Furthermore, miR-21 is also detected in RCC patient serums, and could be used as biomarker but only in combination with other miRNAs, such as miR-106a, miR-310-3p, miR-150-5p and miR-145-5p [90,91,92]. cRCC accounts for 70–85% of all RCC cases, and is typically highly resistant to conventional therapies [93,94]. Studies from the TCGA uncovered that the altered promoter methylation of miR-21 is associated with aggressive cRCC, suggesting that miR-21 may exert an important oncogenic function in this neoplasia [81,95]. miR-21 is not only upregulated in cRCC but is also involved in cancer progression (proliferation, migration, invasion, epithelial mesenchymal transition) and the cancer stem cell phenotype by targeting tumor suppressor genes such as PTEN, PDCD4, TIMP3 or LATS1 [84,85,96,97,98,99,100,101,102,103,104]. As cRCC is typically highly resistant to conventional systemic therapies [93,94], the identification of new molecular mechanisms driving tumor progression is essential for the rational design of new therapeutic strategies to cure cRCC. In this context, miR-21 has been shown to be involved in the resistance to conventional chemotherapies (paclitaxel, 5-Fluorouracil, topotecan and platinum-based therapy) and targeted therapies such as dovitinib and sorafenib by controlling the expression of genes associated with multi-drug resistance (MDR) and the apoptotic pathway (PTEN, PDCD4) [85,105,106]. Similar to renal fibrosis, miR-21 seems to also be involved in the metabolic shift characterizing renal cancer by targeting PPAR-α, a master regulator of lipid metabolism [107]. miR-21 silencing using antisense oligonucleotide or miR-21 sponge strategies decreases the proliferation, invasion and migration of cRCC cells and also increases the expression of pro-apoptotic markers. Furthermore, the inhibition of miR-21 enhances the sensitivity of cRCC cells to conventional genotoxic drugs, as well as to targeted therapies [84,105,106]. Finally, the inhibition of miR-21 also decreases the expression of MDR genes by a mechanism that remains to be deciphered.

## 5. Potential of miR-21 as a Therapeutic Target

The concept of RNA-based therapeutics was conceived over four decades ago when Zamencnik et al. demonstrated that an oligonucleotide directed against the Rous sarcoma virus (RSV) 35S RNA can efficiently inhibit RSV replication [108]. However, the first generation of antisense oligonucleotides (ASOs) showed little therapeutic benefit, given the issues with their short half-lives, suboptimal affinity for mRNA and poor tissue penetration. Recently, the explosion in ncRNA research has renewed interest in developing ASO therapy, and substantial progress has been made in the therapeutic development of ASO with, in particular, cost-effective methods for the synthesis of oligonucleotides and the demonstration that chemical modifications improve the pharmacokinetics properties of these molecules while reducing off-target effects and toxicity. These modifications notably include the use of 2′-O-methyl RNA molecules, the replacement of the phosphodiester backbone with a phosphorothioate backbone, and the use of locked bicyclic nucleic acids [32,109]. Additionally, linking these modified RNA molecules to lipids or other moieties improves delivery by enabling targeting to various tissues or even to specific cell types. Furthermore, modifications of the native RNA backbone reduced the type I interferon response, which is usually induced in response to exposure to extracellular RNA or DNA [32,109]. High concentrations of ASO are usually found in the kidney after systemic administration, in particular in the proximal tubule. Moreover, it has also been shown that the diseased glomerulus also accumulate ASO in all cell types. These findings indicate that this therapeutic strategy may be beneficial in both glomerular and tubulointerstitial diseases [110].

Given that aberrant miRNA expression has been causatively linked to a vast array of chronic diseases, the development of miRNA-based therapies has become a major goal of the current research. Indeed, over the last decade, several miRNA targets have emerged on the basis of strong in vitro and in vivo data, and drugs targeting canonical miRNAs have been developed or are in development to treat specific patient populations with chronic or rare diseases. Of these miRNAs, miR-21 has received particular attention given its abundant baseline expression and strong induction in various physio-pathological conditions, including development, oncology, stem cell biology and ageing. In particular, the successful pharmacological silencing of miR-21 has been achieved in various experimental models of AKI, as well as in chronic glomerular and tubulointerstitial diseases. This includes in particular common kidney disorders such as diabetic nephropathy or renal fibrosis as, well as rare renal diseases such as Alport syndrome [40,69]. For example, the targeting of miR-21 in a genetic rodent model of Alport nephropathy significantly improved albuminuria, renal inflammation, glomerulosclerosis, tubular injury and interstitial fibrosis, as well as survival [75]. Interestingly, these preclinical results gave rise to the development of Lademirsen, an inhibitor of miR-21 currently under investigation for the treatment of Alport syndrome (NCT02855268).

However, the targeting of miR-21 is fraught with many challenges, especially tissue delivery or the determination of the optimal drug concentration to minimize off-target effects without compromising effectiveness. This is of particular importance given the ubiquitous and high expression of miR-21. For example, the systemic delivery of miR-21 ASO-therapy may expose patients to unintended side effects in extrarenal tissues despite an improvement in kidney function. Nevertheless, preliminary results from a phase I clinical trial using miR-21 antagonists in healthy volunteers showed that this drug was well tolerated [111].

## 6. Conclusions

These examples illustrate the complex role of miR-21 during renal insults. They are in favor of a protective role of miR-21 in the initial phase of an aggression (in particular in preconditioning) protecting from subsequent renal damage, but deleterious in chronicity. The exploration of the underlying molecular mechanisms, and in particular the identification of the targets of miR-21, could improve the understanding of the ambivalent role of miR-21. To conclude, although miR-21-5p has an established role in renal disorders, its diagnostic use as biomarker is questionable, given the lack of specificity of its dysregulation. Indeed, it is now well known that dysregulation of miR-21-5p is associated with many renal and extrarenal diseases. Therefore, the therapeutic targeting of miR-21 seems to be a more reasonable approach, and should be the focus of future research studies.

## Supplementary Materials

The following supporting information can be downloaded at: https://www.mdpi.com/article/10.3390/cells11213525/s1, Table S1: implication of miR-21-5p in human renal pathologies; Table S2: implication of miR-21a-5p in animal models of acute and chronic renal pathologies.
