# Supplementary material for: The Versatile Role of miR-21 in Renal Homeostasis and Diseases
Source: Cells. 2022 Nov 7;11(21):3525. doi: 10.3390/cells11213525 (PMC9657972; doi:10.3390/cells11213525)
Supplement: Supplementary file 1 [file cells-11-03525-s001.zip › cells-2000236-supplementary.pdf]

Table S1 : Implication of miR-21-5p in human renal pathology.

Kidney disease	Renal expression of miR-21-5p	Circulating miR-21-5p	Urinary level of miR-21-5p	Other expression of miR-21-5p	References
Acute kidney injury (AKI)			Elevated urinary level of miR-21-5p in AKI patients.<br>No association with AKI no recovery at 90 days.		[1]
		Increased miR-21-5p level after cardiopulmonary bypass surgery with ischemic preconditioning.			[2]
		Increased miR-21-5p level in AKI patients.<br>miR-21-5p associated with AKI clinical stage.	Higher level of miR-21-5p in AKI patients.		[3]
		Lower serum and urinary levels of miR-21-5p in patients with cardiac surgery-associated AKI.			[4]
		Elevated levels of miR-21-5p in acute tubular necrosis and Hepatorenal Syndrom.			[5]
		Decreased circulating miR-21-5p associated with post cardiac surgery AKI			[6]
	Increased renal expression of miR-21-5p in transplanted patients with acute tubular necrosis		Increased urinary level of miR-21-5p after:<br>- acetaminophen-induced AKI<br>- cisplatin-induced AKI		[7]
		Increased urinary and circulating miR-21-5p levels in patients with post cardiac surgery AKI			[8]

			Increased urinary level of miR-21-5p in patients with AKI		[9]
			Increased urinary level of miR-21-5p in patients with AKI		[10]
Nephrolithiasis	Increased miR-21-5p expression		Increased miR-21-5p urinary level		[11]
papillary renal cell carcinoma		Increased miR-21-5p levels			[12]
Nephroangiosclerosis			Increased urinary level of miR-21-5p associated with a better renal outcome		[13]
Diabetic nephropathy			Increased urinary exosomal expression of miR-21-5p associated with:<br>- higher serum creatinine level<br>- lower eGFR		[14]
	Increased miR-21-5p glomerular expression negatively associated with eGFR				[15]
		Increased circulating miR-21-5p level associated with<br>- a low eGFR<br>- a high proteinuria<br>- IRC progression			[16]
		Increased circulating miR-21-5p level associated with renal function worsening			[17]

		Increased circulating miR-21-5p level is not associated with nephropathy			[18]
	Increased renal miR-21-5p level associated with<br>- kidney function worsening<br>- proteinuria<br>- fibrosis (glomerular and interstitial)				[19]
	Increased glomerular miR-21-5p expression associated with proteinuria				[20]
	Increased renal miR-21-5p level				[21]
			Increased urinary levels of miR-21-5p		[22]
			Increased urinary levels of miR-21-5p associated with :<br>- a better renal function<br>- less fibrotic lesions		[23]
			Increased urinary levels of miR-21-5p associated with a better renal outcome		[13]
Kidney transplantation			Increased urinary levels of miR-21-5p in patients with moderate and severe IFTA		[24]
			Increased urinary levels of miR-21-5p in patients with insterstitial fibrosis and tubular atrophy		[25]

				Increased levels of miR-21-5p in hypothermic machine perfusate associated with a worse renal function	[26]
	Increased renal miR-21-3p level in patients developing post-transplant AKI				[27]
	Increased miR-21-5p level in patients with high IF/TA grade	Increased circulating miR-21-5p level in patients with grade 3 IF/TA			[28]
	Increased renal miR-21-5p level associated with graft function, graft survival and renal fibrosis				[29]
IgA nephropathy	Increased miR-21-5p expression				[30]
	Increased miR-21-5p level associated with<br>- glomerular and intestitial fibrosis<br>- renal survival				[31]
	Increased glomerular and tubular miR-21-5p associated with :<br>- glomerular and intestitial fibrosis<br>- proteinuria<br>- renal function				[32]
			Increased urinary levels of miR-21-5p associated with a better renal function		[33]

			Increased urinary levels of miR-21-5p associated with a better renal outcome		[13]
Minimal change nephrotic syndrome			Increased urinary levels of miR-21-5p		[22]
Polycystic Kidney Diseases	miR-21-5p is increased in cyst walls				[34]
Uremic Vascular Calcification		Increased levels of miR-21-5p			[35]
Chronic Kidney Disease			Increased urinary levels of miR-21-5p		[36-37]

AKI : Acute Kidney Injury ; DFG : Débit de filtration glomérulaire ; IF/TA : Interstitial Fibrosis / Tubular Atroph ; eGFR : estimated Glomerular Filtration Rate ; ESRD: End Stage Renal Disease ; CKD: Chronic Kidney Diseease ; VC: Vascular Calcification ; IFTA: Interstitial Fibrosis and Tubular Atrophy

Table S2 : Implication of miR-21a-5p in animal models of acute and chronic renal pathologies.

Kidney disease	miR-21a-5p role	Animal model	Expression of miR-21a-5p	Molecular target : (*cell type* : **miR-21a-5p direct target**)	Référence
Acute Kidney Injury (AKI)	Protective	Cecal ligation and puncture (CLP) in rats<br><br>Overexpression of miR-21a-5p (agomir)	miR-21a-5p ➘ in kidney<br><br><u>Overexpression of miR-21a-5p :</u><br>➚ renal function<br>➘ histological injuries<br>➘ apoptosis, inflammatory and oxidative stress response	*Endothelial progenitor cells (rat) :*<br>**RUNX1**	[38]
		Ischemia-reperfusion mouse model<br><br>Inhibition of miR-21a-5p (KO in dendritic cells)	miR-21a-5p ➘ in dendritic cells<br><br><u>Inhibition of miR-21a-5p :</u><br>➘ renal function<br>➚ histological injuries	*Bone Marrow Derived Dendritic Cell (human) :*<br>**CCR7**	[39]
		Ischemia-reperfusion mouse model<br>Cobalt chloride (protective)<br><br>Inhibition of miR-21a-5p (LNA)	miR-21a-5p ➚ kidney<br><br><u>Inhibition of miR-21a-5p :</u><br>➘ renal function<br>➚ histological injuries	*Umbilical vein endothelial cells (human) :*<br>**thrombospondin-1**	[40]
		Ischemia-reperfusion mouse model<br>Delayed ischemic preconditioning (protective)<br><br>Inhibition of miR-21a-5p (LNA)	miR-21a-5p ➚ whole kidney after preconditioning<br><br><u>Inhibition of miR-21a-5p</u> :<br>Loss of preconditioning benefit (renal function, histology)	*HK2* (Human renal epithelial cells) :<br>**PHD2** – HIF1	[41]
		Ischemia-reperfusion rat model<br>Ghrelin (protective)	miR-21a-5p ➚<br>miR-21a-5p ➚➚ with ghreline	*NRK-52E cells* (Rat renal epithelial cells) :<br>**PTEN** –Akt<br>**Bcl2**	[42]
		Ischemia-reperfusion mouse model<br>Ischemic preconditioning (protective)	miR-21a-5p ➚ 24h after preconditioning	*HEK-296* (human embryonic kidney) and *HK-2 :*	[43]

				**MKK3** – p38<br>MAPK – IL-6/TNF-α	
		Ischemia-reperfusion rat model<br><br>Inhibition of miR-21a-5p (oligonucleotides)	miR-21a-5p ↗ whole kidney<br><br><u>Inhibition of miR-21a-5p</u> :<br>↘ renal function<br>↗ histological injuries	*Cellules NRK-52E* :<br>**Rab11a**	[44]
		Ischemia-reperfusion mouse model,<br>Preconditioning with xenon (protective)<br><br>Inhibition of miR-21a-5p (LNA)	miR-21a-5p ↗ kidney<br>miR-21a-5p ↗↗ with xenon<br><br><u>Inhibition of miR-21a-5p</u> :<br>Loss of xenon benefit (renal function, histology)		[45]
AKI	protective	Injections of gentamycine in rats<br>Preconditioning with xenon (protective)	miR-21a-5p ↗ kidney<br>miR-21a-5p ↗↗ with xenon		[46]
		Ischemia-reperfusion mouse model<br>Ischemic preconditioning (protective)<br><br>Inhibition of miR-21a-5p (LNA)	miR-21a-5p ↗ whole kidney after preconditioning (4h-4 days)<br><br><u>Inhibition of miR-21a-5p</u> :<br>Loss of preconditioning benefit (renal function, histology)<br>No effect in absence of preconditioning	*Epithelial renal cells (human)* :<br>**PDCD4**<br>HIF1	[47]
		Ischemia-reperfusion mouse model	miR-21a-5p ↗ kidney (D1 to D30)	*Tubular cells (mouse)* :<br>**PDCD4**	[48]
	deleterious	Aristolochic acid treated mice<br><br>Inhibition of miR-21a-5p (KO or LNA)	miR-21a-5p ↗ kidney<br><br><u>Inhibition of miR-21a-5p :</u><br>Protection against fibrosis injuries<br>↗ renal function	*HK2 :*<br>**Wnt2b**	[49]
		Ischemia-reperfusion rat model	miR-21a-5p ↗ kidney		[50]

		Vitamin D3 and erythropoietin preconditioning			
		Ischemia-reperfusion mouse model<br>Administration of erythropoietin (protective)	miR-21a-5p ↗ kidney<br>Erythropoietin ↘ miR-21a-5p kidney		[51]
		Cisplatin injections in rat	Urinary miR-21a-5p ↗		[52]
		Ischemia-reperfusion mouse model	miR-21a-5p ↗ kidney<br>miR-21a-5p ↗↗ 24h post reperfusion		[53]
		Ischemia-reperfusion rat model<br><br><br>Gentamycine-induced AKI in rats	miR-21a-5p ↗ cortex ↗↗ medulla<br>↘ miR-21-a-5p in blood<br>↗ miR-21-5p in urine<br><br>miR-21a-5p ↗ cortex and medulla<br>↘ miR-21a-5p in blood and urine		[10]
		Ischemia-reperfusion mouse model<br><br>Inhibition of miR-21a-5p (KO or oligonucleotide)	↗ miR-21a-5p kidney<br><br>Inhibition of miR-21a-5p :<br>Protection against fibrosis injuries	*Myofibrobasts, renal epithelial cells* :<br>ERK/MAPK<br>**PPARα**<br>MPV17-like<br>**RECK**	[54]
		HMOX1$^{-/-}$ mice with low salt diet and injection of Cyclosporin A	miR-21a-5p ↗ whole kidney		[55]
		Low salt diet in mice and injection of Cyclosporin A	miR-21a-5p ↗ whole kidney<br>Up to 2 weeks of treatment then decrease	*Proximal tubular cells (human) :*<br>**PTEN** – Akt	[56]

Renal transplantation	deleterious	Streptozotocin (STZ)-induced diabetic mice<br><br>Inhibition of miR-21a-5p (KO)	miR-21a-5p ↗ kidney<br><br><u>KO miR-21a-5p</u> :<br>↗ proteinuria ↘ renal function<br>↗ glomerulosclerosis	*Podocytes (mice)* :<br>**SMAD7**<br>**PDCD4** p53<br>**TIMP3**	[20]
		db/db mice<br><br>Overexpression of miR-21a-5p (plasmide)	miR-21a-5p ↘ whole kidney<br><br><u>Overexpression of miR-21a-5p</u> :<br>↘ mesangial proliferation<br>↘ albuminuria	*Mesangial cells (mouse) :*<br>**PTEN** - PI3K - Akt	[57]
Diabetic nephropathy (DN)	protective	STZ-induced DN rats<br><br>Inhibition of miR-21a-5p (oligonucleotides)	miR-21a-5p ↗ kidney<br><br><u>Inhibition of miR-21a-5p :</u><br>↘ inflammatory response and progression of the disease<br>↘ renal function	*Podocytes (mouse) :*<br>**TIMP3**	[58]
		KK-Ay mice + high fat diet (diabetus + moderate obesity)	miR-21a-5p ↗ kidney and serum		[59]
	deleterious	STZ-induced diabetic mice<br>Treatment with C66 (curcumin analogue)<br><br>Inhibition of miR-21a-5p (LNA)	C66 ↘ miR-21a-5p kidney<br><br><u>Inhibition of miR-21a-5p :</u><br>- ↘ albuminuria<br>- ↘ renal fibrosis		[60]

		KK-Ay mice + high fat diet (diabetus + moderate obesity)<br><br>Inhibition of miR-21a-5p (antagomir)	miR-21a-5p correlated expression in kidney and sera<br>circulating miR-21a- ↗ associated with :<br>- albuminuria<br>- altered renal function<br>- glomerular injuries<br>Inhibition of miR-21a-5p : improvement of albuminuria, renal function and glomerular injuries	*HKC (Human Kidney Cells)* :<br>**SMAD 7**	[61]
		KK-Ay mice + high fat diet<br><br>Inhibition of miR-21a-5p (lentivirus)	miR-21a-5p ↗ whole kidney<br>Inhibition of miR-21a-5p :<br>- ↘ EMT and fibrosis markers<br>- ↘ albuminuria	*HKC (Human Kidney Cells)* + TGF-ß:<br>TGF-ß – SMAD 3 / **SMAD 7**<br>α-SMA, E-cadherin	[61]
		KK-Ay mice<br><br>Inhibition of miR-21a-5p (oligonucleotide)	miR-21a-5p ↗ whole kidney<br>associated with à albuminuria, renal failure and glomerular sclerosis<br>Inhibition of miR-21a-5p :<br>↘ injuries		[62]
		Streptozotocin-induced diabetic mice<br>TIMP3$^{-/-}$ mice	miR-21a-5p ↗ whole kidney	*Mesangial cells (mice)* :<br>**TIMP3**<br>SirT1	[21]
	Deleterious	db/db mice<br><br>Inhibition of miR-21a-5p (ultrasound-microbubble-mediated miR-21 small hairpin RNA transfer)	miR-21a-5p ↗ whole kidney<br><br>inhibition of miR-21a-5p :<br>- improvement of renal function<br>- inhibition of renal fibrosis and inflammation	*Tubular and mesangial cellsn (rat) :*<br>**SMAD 7**<br>TGF-ß<br>NFκB	[63]

		OVE26 mice (Type 1 diabetus)	miR-21a-5p ↗ renal cortex	*Mesangial cells (rat and human)*:<br>**PTEN** – AKT – GSK3ß - Tor complex 1	[64]
		NOD mice (Non Obese Diabetic mice) PDCD4$^{-/-}$ (type 1 diabetus)	miR-21a-5p ↗ ß islands	*ß cells (mice)* :<br>**PDCD4** – NFκB	[65]
		Col4a3 $^{-/-}$ mice<br><br>Inhibition of miR-21a-5p (oligonucleotide)	miR-21a-5p ↗ kidney<br><br><u>Inhibition of miR-21a-5p</u> :<br>Protection against disease progression and improvement of survival<br>↘ albuminuria<br>↘ expression markers of kidney injury, fibrosis, inflammation, mitochondrial function and lipid metabolism		[32]
Alport nephropathy	deleterious	Col4a3 $^{-/-}$ mice<br><br>Inhibition of miR-21a-5p (oligonucleotide)	miR-21a-5p ↗ whole kidney<br><br><u>Inhibition of miR-21a-5p</u> :<br>Improvement of renal function and proteinuria<br>Survival increased<br>↘ glomerular and interstitial fibrosis	*Proximal tubular cells, podocytes, fibroblasts (mice and human) :*<br>**PPAR-α** – RXR (Retinoid X Receptor)	[66]
Glomerulonephritis	deleterious	Nephrotoxic serum injections in rat (contains antibodies against the glomerular basement membrane)	Urinary miR-21a-5p ↗		[67]
		Glomerulonephritis induced by anti-Thy 1.1 in rats	miR-21a-5p ↗ kidney		[68]

				Circulating T cells (human lupus disease) :<br>**PDCD4**	[69]
Polycystic renal disease	deleterious	Kif3a$^{-/-}$ mice<br>Pkd1$^{-/-}$ mice<br>Pkd2$^{-/-}$ mice<br>Hnf-1ß$^{-/-}$ mice<br>miR-21$^{-/-}$ mice	miR-21a-5p ↗ whole kidney and cysts<br><u>KO miR-21a-5p</u> :<br>↘ cysts<br>Improvement of renal function	*Renal epithelial cells (mice)* :<br>**PDCD4**	[70]
Kidney Stone Disease	deleterious	Calcium oxalate crystal deposition induced by injection of glyoxylate in mice<br><br>Inhibition of miR-21a-5p (oligonucleotides)	miR-21a-5p ↗ kidney<br><br><u>Inhibition of miR-21a-5p</u> :<br>↘ creatinemia<br>↘ renal tubular injury<br>↘ lipid accumulation	*HK2 :*<br>**PPARα**	[34]
Renal fibrosis / Chronic kidney disease	deleterious	TGF-ß transgenic mice (model of glomerular sclerosis)<br><br>Inhibition of miR-21a-5p (KO)	miR-21a-5p ↗ kidney<br><br><u>KO miR-21a-5p</u> :<br>↗ proteinuria<br>↗ glomerusclerosis	*Podocytes (mice)* :<br>**SMAD7**<br>**PDCD4**<br>**p53**<br>**TIMP3**	[11]
		UUO mice	miR-21a-5p ↗ kidney	*Epithelial renal cells (mice) :*<br>SPRY1/ERK/NFκB signalling pathway	[20]
		Angiotensin II-treated mice<br><br>Inhibition of miR-21a-5p (lentivirus)	↗ miR-21a-5p cortical kidney<br><br><u>Inhibition of miR-21a-5p</u> :<br>↘ albuminuria<br>Protection against fibrosis	*HK2 :*<br>**PPARα**	[71]

	deleterious	UUO mice	↗ miR-21a-5p kidney	*HK2 :*<br>ERK1/2	[72]
		Adenine-induced renal fibrosis mouse model	↗ miR-21a-5p cortical kidney		[73]
		Unilateral ureteral obstruction mice (UUO)	↗ miR-21a-5p kidney D4-D28	*Fibroblasts (mice)* :<br>α-SMA, collagen 1A1	[19]
		UUO mice<br><br>Inhibition of miR-21a-5p (KO or oligonucleotide)	↗ miR-21a-5p kidney<br><br>Inhibition of miR-21a-5p :<br>Protection against fibrosis	*Myofibrobases, renal epithelial cells (mice)* :<br>ERK/MAPK<br>**PPARα**<br>MPV17<br>**RECK**	[28]
		UUO mice<br>SMAD3$^{-/-}$ (protective)<br><br>Inhibition of miR-21a-5p (shRNA)	↗ miR-21a-5p kidney<br>↘ miR-21a-5p kidney SMAD3$^{-/-}$ with UUO<br><br>Inhibition of miR-21a-5p :<br>Improvement of histological injuries		[54]
		UUO mice<br><br>Inhibition of miR-21a-5p (LNA)	↗ miR-21a-5p- kidney<br><br>Inhibition of miR-21a-5p :<br>Improvement of histological injuries		[75]
		UUO rats<br>SHRSP rats (hypertensive rats)	miR-21a-5p ↗ kidney		[76]

Abbreviations : AKI : Acute Kidney Injury ; CLP : cecal ligation and puncture ; DN : diabetic nephropathy ; HKC : Human Kidney Cells ; KO : Knock Out ; LNA : Locked Nucleic Acid ; UUO : Ureteral Unilateral Obstruction; shRNA : small hairpin RNA, SHRSP : Spontaneously hypertensive rat stroke-prone ; STZ : Streptozotocin

REFERENCES

1. Newbury LJ, Simpson K, Khalid U, John I, de Rivera LB, Lu YA, Lopez-Anton M, Watkins WJ, Jenkins RH, Fraser DJ, Bowen T. miR-141 mediates recovery from acute kidney injury. Sci Rep. 2021 Aug 13;11(1):16499. doi: 10.1038/s41598-021-94984-x.

2. Kang Z, Li Z, Huang P, Luo J, Liu P, Wang Y, Xia T, Zhou Y. Remote ischemic preconditioning upregulates microRNA-21 to protect the kidney in children with congenital heart disease undergoing cardiopulmonary bypass. Pediatr Nephrol. 2018 May;33(5):911-919. doi: 10.1007/s00467-017-3851-9. Epub 2017 Dec 2. PMID: 29197999.

3. Ma Y, Fu J, Qian L, Tao T. Serum miRNA expression and correlation with clinical characteristics in acute kidney injury. Int J Clin Exp Pathol. 2017 Aug 1;10(8):8721-8726.

4. Arvin P, Samimagham HR, Montazerghaem H, Khayatian M, Mahboobi H, Ghadiri Soufi F. Early detection of cardiac surgery-associated acute kidney injury by microRNA-21. Bratisl Lek Listy. 2017;118(10):626-631. doi: 10.4149/BLL_2017_120.

5. Watany MM, Hagag RY, Okda HI. Circulating miR-21, miR-210 and miR-146a as potential biomarkers to differentiate acute tubular necrosis from hepatorenal syndrome in patients with liver cirrhosis: a pilot study. Clin Chem Lab Med. 2018 Apr 25;56(5):739-747. doi: 10.1515/cclm-2017-0483.

6. Gaede L, Liebetrau C, Blumenstein J, Troidl C, Dörr O, Kim WK, Gottfried K, Voss S, Berkowitsch A, Walther T, Nef H, Hamm CW, Möllmann H. Plasma microRNA-21 for the early prediction of acute kidney injury in patients undergoing major cardiac surgery. Nephrol Dial Transplant. 2016 May;31(5):760-6. doi: 10.1093/ndt/gfw007.

7. Pavkovic M, Robinson-Cohen C, Chua AS, Nicoara O, Cárdenas-González M, Bijol V, Ramachandran K, Hampson L, Pirmohamed M, Antoine DJ, Frendl G, Himmelfarb J, Waikar SS, Vaidya VS. Detection of Drug-Induced Acute Kidney Injury in Humans Using Urinary KIM-1, miR-21, -200c, and -423. Toxicol Sci. 2016 Jul;152(1):205-13. doi: 10.1093/toxsci/kfw077.

8. Du J, Cao X, Zou L, Chen Y, Guo J, Chen Z, Hu S, Zheng Z. MicroRNA-21 and risk of severe acute kidney injury and poor outcomes after adult cardiac surgery. PLoS One. 2013 May 23;8(5):e63390. doi: 10.1371/journal.pone.0063390.

9. Ramachandran K, Saikumar J, Bijol V, Koyner JL, Qian J, Betensky RA, Waikar SS, Vaidya VS. Human miRNome profiling identifies microRNAs differentially present in the urine after kidney injury. Clin Chem. 2013 Dec;59(12):1742-52. doi: 10.1373/clinchem.2013.210245.

10. Saikumar J, Hoffmann D, Kim TM, Gonzalez VR, Zhang Q, Goering PL, Brown RP, Bijol V, Park PJ, Waikar SS, Vaidya VS. Expression, circulation, and excretion profile of microRNA-21, -155, and -18a following acute kidney injury. Toxicol Sci. 2012 Oct;129(2):256-67. doi: 10.1093/toxsci/kfs210.

11. Su B, Han H, Ji C, Hu W, Yao J, Yang J, Fan Y, Li J. MiR-21 promotes calcium oxalate-induced renal tubular cell injury by targeting PPARA. Am J Physiol Renal Physiol. 2020 Aug 1;319(2):F202-F214. doi: 10.1152/ajprenal.00132.2020.

12. Kalogirou C, Ellinger J, Kristiansen G, Hatzichristodoulou G, Kübler H, Kneitz B, Busch J, Fendler A. Identification of miR-21-5p and miR-210-3p serum levels as biomarkers for patients with papillary renal cell carcinoma: a multicenter analysis. Transl Androl Urol. 2020 Jun;9(3):1314-1322. doi: 10.21037/tau.2020.03.18.

13. Szeto CC, Ching-Ha KB, Ka-Bik L, Mac-Moune LF, Cheung-Lung CP, Gang W, Kai-Ming C, Kam-Tao LP. Micro-RNA expression in the urinary sediment of patients with chronic kidney diseases. Dis Markers. 2012;33(3):137-44. doi: 10.1155/2012/842764.

14. Zang J, Maxwell AP, Simpson DA, McKay GJ. Differential Expression of Urinary Exosomal MicroRNAs miR-21-5p and miR-30b-5p in Individuals with Diabetic Kidney Disease. Sci Rep. 2019 Jul 29;9(1):10900. doi: 10.1038/s41598-019-47504-x.

15. Baker MA, Davis SJ, Liu P, Pan X, Williams AM, Iczkowski KA, Gallagher ST, Bishop K, Regner KR, Liu Y, Liang M. Tissue-Specific MicroRNA Expression Patterns in Four Types of Kidney Disease. J Am Soc Nephrol. 2017 Oct;28(10):2985-2992. doi: 10.1681/ASN.2016121280.

16. Chien HY, Chen CY, Chiu YH, Lin YC, Li WC. Differential microRNA Profiles Predict Diabetic Nephropathy Progression in Taiwan. Int J Med Sci. 2016 Jun 1;13(6):457-65. doi: 10.7150/ijms.15548.

17. Pezzolesi MG, Satake E, McDonnell KP, Major M, Smiles AM, Krolewski AS. Circulating TGF-β1-Regulated miRNAs and the Risk of Rapid Progression to ESRD in Type 1 Diabetes. Diabetes. 2015 Sep;64(9):3285-93. doi: 10.2337/db15-0116.

18. Olivieri F, Spazzafumo L, Bonafè M, Recchioni R, Prattichizzo F, Marcheselli F, Micolucci L, Mensà E, Giuliani A, Santini G, Gobbi M, Lazzarini R, Boemi M, Testa R, Antonicelli R, Procopio AD, Bonfigli AR. MiR-21-5p and miR-126a-3p levels in plasma and circulating angiogenic cells: relationship with type 2 diabetes complications. Oncotarget. 2015 Nov 3;6(34):35372-82. doi: 10.18632/oncotarget.6164.

19. McClelland AD, Herman-Edelstein M, Komers R, Jha JC, Winbanks CE, Hagiwara S, Gregorevic P, Kantharidis P, Cooper ME. miR-21 promotes renal fibrosis in diabetic nephropathy by targeting PTEN and SMAD7. Clin Sci (Lond). 2015 Dec;129(12):1237-49. doi: 10.1042/CS20150427.

20. Lai JY, Luo J, O'Connor C, Jing X, Nair V, Ju W, Randolph A, Ben-Dov IZ, Matar RN, Briskin D, Zavadil J, Nelson RG, Tuschl T, Brosius FC 3rd, Kretzler M, Bitzer M. MicroRNA-21 in glomerular injury. J Am Soc Nephrol. 2015 Apr;26(4):805-16. doi: 10.1681/ASN.2013121274.

21. Fiorentino L, Cavalera M, Mavilio M, Conserva F, Menghini R, Gesualdo L, Federici M. Regulation of TIMP3 in diabetic nephropathy: a role for microRNAs. Acta Diabetol. 2013 Dec;50(6):965-9. doi: 10.1007/s00592-013-0492-8.

22. Konta T, Ichikawa K, Suzuki K, Kudo K, Satoh H, Kamei K, Nishidate E, Kubota I. A microarray analysis of urinary microRNAs in renal diseases. Clin Exp Nephrol. 2014 Oct;18(5):711-7. doi: 10.1007/s10157-013-0906-5.

23. Wang J, Duan L, Tian L, Liu J, Wang S, Gao Y, Yang J. Serum miR-21 may be a Potential Diagnostic Biomarker for Diabetic Nephropathy. Exp Clin Endocrinol Diabetes. 2016 Jul;124(7):417-23. doi: 10.1055/s-0035-1565095. Epub 2015 Nov 17. PMID: 26575121.

24. Gniewkiewicz MS, Paszkowska I, Gozdowska J, Czerwinska K, Sadowska-Jakubowicz A, Deborska-Materkowska D, Perkowska-Ptasinska A, Kosieradzki M, Durlik M. Urinary MicroRNA-21-5p as Potential Biomarker of Interstitial Fibrosis and Tubular Atrophy (IFTA) in Kidney Transplant Recipients. Diagnostics (Basel). 2020 Feb 19;10(2):113. doi: 10.3390/diagnostics10020113.

25. Zununi Vahed S, Omidi Y, Ardalan M, Samadi N. Dysregulation of urinary miR-21 and miR-200b associated with interstitial fibrosis and tubular atrophy (IFTA) in renal transplant recipients. Clin Biochem. 2017 Jan;50(1-2):32-39. doi: 10.1016/j.clinbiochem.2016.08.007.

26. Khalid U, Ablorsu E, Szabo L, Jenkins RH, Bowen T, Chavez R, Fraser DJ. MicroRNA-21 (miR-21) expression in hypothermic machine perfusate may be predictive of early outcomes in kidney transplantation. Clin Transplant. 2016 Feb;30(2):99-104. doi: 10.1111/ctr.12679.

27. Wilflingseder J, Sunzenauer J, Toronyi E, Heinzel A, Kainz A, Mayer B, Perco P, Telkes G, Langer RM, Oberbauer R. Molecular pathogenesis of post-transplant acute kidney injury: assessment of whole-genome mRNA and miRNA profiles. PLoS One. 2014 Aug 5;9(8):e104164. doi: 10.1371/journal.pone.0104164.

28. Glowacki F, Savary G, Gnemmi V, Buob D, Van der Hauwaert C, Lo-Guidice JM, Bouyé S, Hazzan M, Pottier N, Perrais M, Aubert S, Cauffiez C. Increased circulating miR-21 levels are associated with kidney fibrosis. PLoS One. 2013;8(2):e58014. doi: 10.1371/journal.pone.0058014.

29. Ben-Dov IZ, Muthukumar T, Morozov P, Mueller FB, Tuschl T, Suthanthiran M. MicroRNA sequence profiles of human kidney allografts with or without tubulointerstitial fibrosis. Transplantation. 2012 Dec 15;94(11):1086-94. doi: 10.1097/TP.0b013e3182751efd.

30. Szeto CC, Ng JK, Fung WW, Luk CC, Wang G, Chow KM, Lai KB, Li PK, Lai FM. Kidney microRNA-21 Expression and Kidney Function in IgA Nephropathy. Kidney Med. 2020 Dec 4;3(1):76-82.e1. doi: 10.1016/j.xkme.2020.11.009.

31. Hennino MF, Buob D, Van der Hauwaert C, Gnemmi V, Jomaa Z, Pottier N, Savary G, Drumez E, Noël C, Cauffiez C, Glowacki F. miR-21-5p renal expression is associated with fibrosis and renal survival in patients with IgA nephropathy. Sci Rep. 2016 Jun 6;6:27209. doi: 10.1038/srep27209.

32. Bao H, Hu S, Zhang C, Shi S, Qin W, Zeng C, Zen K, Liu Z. Inhibition of miRNA-21 prevents fibrogenic activation in podocytes and tubular cells in IgA nephropathy. Biochem Biophys Res Commun. 2014 Feb 21;444(4):455-60. doi: 10.1016/j.bbrc.2014.01.065. Epub 2014 Jan 24. PMID: 24468088.

33. Wang G, Kwan BC, Lai FM, Chow KM, Kam-Tao Li P, Szeto CC. Expression of microRNAs in the urinary sediment of patients with IgA nephropathy. Dis Markers. 2010;28(2):79-86. doi: 10.3233/DMA-2010-0687.

34. Lakhia R, Hajarnis S, Williams D, Aboudehen K, Yheskel M, Xing C, Hatley ME, Torres VE, Wallace DP, Patel V. MicroRNA-21 Aggravates Cyst Growth in a Model of Polycystic Kidney Disease. J Am Soc Nephrol. 2016 Aug;27(8):2319-30. doi: 10.1681/ASN.2015060634.

35. Wu R, Zhou S, Liu M, An H, Wang Z, Liu T. Clinical Significance of miR-21-5p in Predicting Occurrence and Progression of Uremic Vascular Calcification in Patients with End-Stage Renal Disease. Yonsei Med J. 2022 Mar;63(3):252-258. doi: 10.3349/ymj.2022.63.3.252.

36. Lange T, Artelt N, Kindt F, Stracke S, Rettig R, Lendeckel U, Chadjichristos CE, Kavvadas P, Chatziantoniou C, Endlich K, Endlich N. MiR-21 is up-regulated in urinary exosomes of chronic kidney disease patients and after glomerular injury. J Cell Mol Med. 2019 Jul;23(7):4839-4843. doi: 10.1111/jcmm.14317.

37. Donderski R, Szczepanek J, Naruszewicz N, Naruszewicz R, Tretyn A, Skoczylas-Makowska N, Tyloch J, Odrowąż-Sypniewska G, Manitius J. Analysis of profibrogenic microRNAs (miRNAs) expression in urine and serum of chronic kidney disease (CKD) stage 1-4 patients and their relationship with proteinuria and kidney function. Int Urol Nephrol. 2022 Apr;54(4):937-947. doi: 10.1007/s11255-021-02928-1.

38. Zhang Y, Huang H, Liu W, Liu S, Wang XY, Diao ZL, Zhang AH, Guo W, Han X, Dong X, Katilov O. Endothelial progenitor cells-derived exosomal microRNA-21-5p alleviates sepsis-induced acute kidney injury by inhibiting RUNX1 expression. Cell Death Dis. 2021 Mar 30;12(4):335. doi: 10.1038/s41419-021-03578-y.

39. Jia P, Pan T, Xu S, Fang Y, Song N, Guo M, Liang Y, Xu X, Ding X. Depletion of miR-21 in dendritic cells aggravates renal ischemia-reperfusion injury. FASEB J. 2020 Sep;34(9):11729-11740. doi: 10.1096/fj.201903222RR.

40. Xu X, Song N, Zhang X, Jiao X, Hu J, Liang M, Teng J, Ding X. Renal Protection Mediated by Hypoxia Inducible Factor-1α Depends on Proangiogenesis Function of miR-21 by Targeting Thrombospondin 1. Transplantation. 2017 Aug;101(8):1811-1819. doi: 10.1097/TP.0000000000001501.

41. Jiao X, Xu X, Fang Y, Zhang H, Liang M, Teng J, Ding X. miR-21 contributes to renal protection by targeting prolyl hydroxylase domain protein 2 in delayed ischaemic preconditioning. Nephrology (Carlton). 2017 May;22(5):366-373. doi: 10.1111/nep.12787.

42. Zhang W, Shu L. Upregulation of miR-21 by Ghrelin Ameliorates Ischemia/Reperfusion-Induced Acute Kidney Injury by Inhibiting Inflammation and Cell Apoptosis. DNA Cell Biol. 2016 Aug;35(8):417-25. doi: 10.1089/dna.2016.3231.

43. Li Z, Deng X, Kang Z, Wang Y, Xia T, Ding N, Yin Y. Elevation of miR-21, through targeting MKK3, may be involved in ischemia pretreatment protection from ischemia-reperfusion induced kidney injury. J Nephrol. 2016 Feb;29(1):27-36. doi: 10.1007/s40620-015-0217-x.

44. Liu X, Hong Q, Wang Z, Yu Y, Zou X, Xu L. MiR-21 inhibits autophagy by targeting Rab11a in renal ischemia/reperfusion. Exp Cell Res. 2015 Oct 15;338(1):64-9. doi: 10.1016/j.yexcr.2015.08.010.

45. Jia P, Teng J, Zou J, Fang Y, Zhang X, Bosnjak ZJ, Liang M, Ding X. miR-21 contributes to xenon-conferred amelioration of renal ischemia-reperfusion injury in mice. Anesthesiology. 2013 Sep;119(3):621-30. doi: 10.1097/ALN.0b013e318298e5f1.

46. Jia P, Teng J, Zou J, Fang Y, Jiang S, Yu X, Kriegel AJ, Liang M, Ding X. Intermittent exposure to xenon protects against gentamicin-induced nephrotoxicity. PLoS One. 2013 May 30;8(5):e64329. doi: 10.1371/journal.pone.0064329.

47. Xu X, Kriegel AJ, Liu Y, Usa K, Mladinov D, Liu H, Fang Y, Ding X, Liang M. Delayed ischemic preconditioning contributes to renal protection by upregulation of miR-21. Kidney Int. 2012 Dec;82(11):1167-75. doi: 10.1038/ki.2012.241.

48. Godwin JG, Ge X, Stephan K, Jurisch A, Tullius SG, Iacomini J. Identification of a microRNA signature of renal ischemia reperfusion injury. Proc Natl Acad Sci U S A. 2010 Aug 10;107(32):14339-44. doi: 10.1073/pnas.0912701107.

49. Kuang Q, Wu S, Xue N, Wang X, Ding X, Fang Y. Selective Wnt/beta-Catenin Pathway Activation Concomitant With Sustained Overexpression of miR-21 is Responsible for Aristolochic Acid-Induced AKI-to-CKD Transition. Front Pharmacol. 2021 May 28;12:667282. doi: 10.3389/fphar.2021.667282.

50. Golmohammadi MG, Banaei S, Nejati K, Chinifroush-Asl MM. Vitamin D3 and erythropoietin protect against renal ischemia-reperfusion injury via heat shock protein 70 and microRNA-21 expression. Sci Rep. 2020 Dec 1;10(1):20906. doi: 10.1038/s41598-020-78045-3.

51. Chen X, Wang CC, Song SM, Wei SY, Li JS, Zhao SL, Li B. The administration of erythropoietin attenuates kidney injury induced by ischemia/reperfusion with increased activation of Wnt/β-catenin signaling. J Formos Med Assoc. 2015 May;114(5):430-7. doi: 10.1016/j.jfma.2015.01.007.

52. Pavkovic M, Riefke B, Ellinger-Ziegelbauer H. Urinary microRNA profiling for identification of biomarkers after cisplatin-induced kidney injury. Toxicology. 2014 Oct 3;324:147-57. doi: 10.1016/j.tox.2014.05.005.

53. Kaucsár T, Révész C, Godó M, Krenács T, Albert M, Szalay CI, Rosivall L, Benyó Z, Bátkai S, Thum T, Szénási G, Hamar P. Activation of the miR-17 family and miR-21 during murine kidney ischemia-reperfusion injury. Nucleic Acid Ther. 2013 Oct;23(5):344-54. doi: 10.1089/nat.2013.0438.

54. Chau BN, Xin C, Hartner J, Ren S, Castano AP, Linn G, Li J, Tran PT, Kaimal V, Huang X, Chang AN, Li S, Kalra A, Grafals M, Portilla D, MacKenna DA, Orkin SH, Duffield JS. MicroRNA-21 promotes fibrosis of the kidney by silencing metabolic pathways. Sci Transl Med. 2012 Feb 15;4(121):121ra18. doi: 10.1126/scitranslmed.3003205.

55. Łoboda A, Mucha O, Podkalicka P, Sobczak M, Miksza-Cybulska A, Kaczara P, Jozkowicz A, Dulak J. Kidney injury by cyclosporine A is aggravated in heme oxygenase-1 deficient

mice and involves regulation of microRNAs. Acta Biochim Pol. 2018 Nov 27;65(4):613-620. doi: 10.18388/abp.2018_2658.

56. Chen J, Zmijewska A, Zhi D, Mannon RB. Cyclosporine-mediated allograft fibrosis is associated with micro-RNA-21 through AKT signaling. Transpl Int. 2015 Feb;28(2):232-45. doi: 10.1111/tri.12471.

57. Zhang Z, Peng H, Chen J, Chen X, Han F, Xu X, He X, Yan N. MicroRNA-21 protects from mesangial cell proliferation induced by diabetic nephropathy in db/db mice. FEBS Lett. 2009 Jun 18;583(12):2009-14. doi: 10.1016/j.febslet.2009.05.021.

58. Chen X, Zhao L, Xing Y, Lin B. Down-regulation of microRNA-21 reduces inflammation and podocyte apoptosis in diabetic nephropathy by relieving the repression of TIMP3 expression. Biomed Pharmacother. 2018 Dec;108:7-14. doi: 10.1016/j.biopha.2018.09.007.

59. Wang X, Gao Y, Tian N, Zou D, Shi Y, Zhang N. Astragaloside IV improves renal function and fibrosis via inhibition of miR-21-induced podocyte dedifferentiation and mesangial cell activation in diabetic mice. Drug Des Devel Ther. 2018 Aug 6;12:2431-2442. doi: 10.2147/DDDT.S170840.

60. Wu H, Kong L, Tan Y, Epstein PN, Zeng J, Gu J, Liang G, Kong M, Chen X, Miao L, Cai L. C66 ameliorates diabetic nephropathy in mice by both upregulating NRF2 function via increase in miR-200a and inhibiting miR-21. Diabetologia. 2016 Jul;59(7):1558-1568. doi: 10.1007/s00125-016-3958-8.

61. Wang JY, Gao YB, Zhang N, Zou DW, Wang P, Zhu ZY, Li JY, Zhou SN, Wang SC, Wang YY, Yang JK. miR-21 overexpression enhances TGF-β1-induced epithelial-to-mesenchymal transition by target smad7 and aggravates renal damage in diabetic nephropathy. Mol Cell Endocrinol. 2014 Jul 5;392(1-2):163-72. doi: 10.1016/j.mce.2014.05.018.

62. Wang J, Gao Y, Ma M, Li M, Zou D, Yang J, Zhu Z, Zhao X. Effect of miR-21 on renal fibrosis by regulating MMP-9 and TIMP1 in kk-ay diabetic nephropathy mice. Cell Biochem Biophys. 2013 Nov;67(2):537-46. doi: 10.1007/s12013-013-9539-2. PMID: 23443810.

63. Zhong X, Chung AC, Chen HY, Dong Y, Meng XM, Li R, Yang W, Hou FF, Lan HY. miR-21 is a key therapeutic target for renal injury in a mouse model of type 2 diabetes. Diabetologia. 2013 Mar;56(3):663-74. doi: 10.1007/s00125-012-2804-x.

64. Dey N, Ghosh-Choudhury N, Kasinath BS, Choudhury GG. TGFβ-stimulated microRNA-21 utilizes PTEN to orchestrate AKT/mTORC1 signaling for mesangial cell hypertrophy and matrix expansion. PLoS One. 2012;7(8):e42316. doi: 10.1371/journal.pone.0042316.

65. Ruan Q, Wang T, Kameswaran V, Wei Q, Johnson DS, Matschinsky F, Shi W, Chen YH. The microRNA-21-PDCD4 axis prevents type 1 diabetes by blocking pancreatic beta cell death. Proc Natl Acad Sci U S A. 2011 Jul 19;108(29):12030-5. doi: 10.1073/pnas.1101450108.

66. Guo J, Song W, Boulanger J, Xu EY, Wang F, Zhang Y, He Q, Wang S, Yang L, Pryce C, Phillips L, MacKenna D, Leberer E, Ibraghimov-Beskrovnaya O, Ding J, Liu S. Dysregulated

Expression of microRNA-21 and Disease-Related Genes in Human Patients and in a Mouse Model of Alport Syndrome. Hum Gene Ther. 2019 Jul;30(7):865-881. doi: 10.1089/hum.2018.205.

67. Gomez IG, MacKenna DA, Johnson BG, Kaimal V, Roach AM, Ren S, Nakagawa N, Xin C, Newitt R, Pandya S, Xia TH, Liu X, Borza DB, Grafals M, Shankland SJ, Himmelfarb J, Portilla D, Liu S, Chau BN, Duffield JS. Anti-microRNA-21 oligonucleotides prevent Alport nephropathy progression by stimulating metabolic pathways. J Clin Invest. 2015 Jan;125(1):141-56. doi: 10.1172/JCI75852.

68. Pavkovic M, Riefke B, Frisk AL, Gröticke I, Ellinger-Ziegelbauer H. Glomerulonephritis-Induced Changes in Urinary and Kidney MicroRNA Profiles in Rats. Toxicol Sci. 2015 Jun;145(2):348-59. doi: 10.1093/toxsci/kfv053.

69. Denby L, Ramdas V, McBride MW, Wang J, Robinson H, McClure J, Crawford W, Lu R, Hillyard DZ, Khanin R, Agami R, Dominiczak AF, Sharpe CC, Baker AH. miR-21 and miR-214 are consistently modulated during renal injury in rodent models. Am J Pathol. 2011 Aug;179(2):661-72. doi: 10.1016/j.ajpath.2011.04.021.

70. Stagakis E, Bertsias G, Verginis P, Nakou M, Hatziapostolou M, Kritikos H, Iliopoulos D, Boumpas DT. Identification of novel microRNA signatures linked to human lupus disease activity and pathogenesis: miR-21 regulates aberrant T cell responses through regulation of PDCD4 expression. Ann Rheum Dis. 2011 Aug;70(8):1496-506. doi: 10.1136/ard.2010.139857.

71. Liu E, Lv L, Zhan Y, Ma Y, Feng J, He Y, Wen Y, Zhang Y, Pu Q, Ji F, Yang X, Wen JG. METTL3/N6-methyladenosine/ miR-21-5p promotes obstructive renal fibrosis by regulating inflammation through SPRY1/ERK/NF-κB pathway activation. J Cell Mol Med. 2021 Aug;25(16):7660-7674. doi: 10.1111/jcmm.16603. Epub 2021 Jun 24. PMID: 34164910; PMCID: PMC8358893.

72. Masum MA, Ichii O, Elewa YHA, Kon Y. Podocyte Injury Through Interaction Between Tlr8 and Its Endogenous Ligand miR-21 in Obstructed and Its Collateral Kidney. Front Immunol. 2021 Jan 22;11:606488. doi: 10.3389/fimmu.2020.606488.

73. Lyu H, Li X, Wu Q, Hao L. Overexpression of microRNA-21 mediates Ang II-induced renal fibrosis by activating the TGF-beta1/Smad3 pathway via suppressing PPARalpha. Pharmacol Sci. 2019 Sep;141(1):70-78. doi: 10.1016/j.jphs.2019.09.007.

74. Tang CR, Luo SG, Lin X, Wang J, Liu Y. Silenced miR-21 inhibits renal interstitial fibrosis via targeting ERK1/2 signaling pathway in mice. Eur Rev Med Pharmacol Sci. 2019 Aug;23(3 Suppl):110-116. doi: 10.26355/eurrev_201908_18637.

75. Zhong X, Chung AC, Chen HY, Meng XM, Lan HY. Smad3-mediated upregulation of miR-21 promotes renal fibrosis. J Am Soc Nephrol. 2011 Sep;22(9):1668-81. doi: 10.1681/ASN.2010111168.

76. Zarjou A, Yang S, Abraham E, Agarwal A, Liu G. Identification of a microRNA signature in renal fibrosis: role of miR-21. Am J Physiol Renal Physiol. 2011 Oct;301(4):F793-801. doi: 10.1152/ajprenal.00273.2011.
